# The re-enactment of childhood sexual abuse in maternity care: a qualitative study

**DOI:** 10.1186/s12884-015-0626-9

**Published:** 2015-08-26

**Authors:** Elsa Montgomery, Catherine Pope, Jane Rogers

**Affiliations:** Florence Nightingale Faculty of Nursing and Midwifery, James Clerk Maxwell Building, 57 Waterloo Road, London, SEI 8WA UK; Faculty of Health Sciences, Highfield Campus, University Road, Southampton, SO17 1BJ UK; Princess Anne Hospital, Coxford Road, Southampton, SO16 5YA UK

## Abstract

**Background:**

The process of pregnancy and birth are profound events that can be particularly challenging for women with a history of childhood sexual abuse. The silence that surrounds childhood sexual abuse means that few women disclose it and those caring for them will often not be aware of their history. It is known from anecdotal accounts that distressing memories may be triggered by childbirth and maternity care but research data on the subject are rare. This paper explores aspects of a study on the maternity care experiences of women who were sexually abused in childhood that demonstrate ways that maternity care can be reminiscent of abuse. Its purpose is to inform those providing care for these women.

**Methods:**

The experiences of women were explored through in-depth interviews in this feminist narrative study. The Voice-Centred Relational Method and thematic analysis were employed to examine interview data.

**Results:**

Women sometimes experienced re-enactment of abuse through intimate procedures but these were not necessarily problematic in themselves. How they were conducted was important. Women also experienced re-enactment of abuse through pain, loss of control, encounters with strangers and unexpected triggers. Many of these experiences were specific to the woman, often unpredictable and not necessarily avoidable. Maternity care was reminiscent of abuse for women irrespective of whether they had disclosed to midwives and was not necessarily prevented by sensitive care. ‘Re-enactment of abuse’ occurred both as a result of events that involved the crossing of a woman’s body boundaries and more subjective internal factors that related to her sense of agency.

**Conclusions:**

As staff may not know of a woman’s history, they must be alert to unspoken messages and employ ‘universal precautions’ to mitigate hidden trauma. Demonstrating respect and enabling women to retain control is crucial. Getting to know women is important in the building of trusting relationships that will facilitate the delivery of sensitive care and enable women to feel safe so that the re-enactment of abuse in maternity care is minimised.

## Background

Pregnancy and birth are profound events in any woman’s life [1, 2] but for some navigation through the process can be particularly challenging. This includes women whose previous life experiences potentially make the encounters involved in becoming a mother very difficult such as those with a history of childhood sexual abuse (CSA). By its very nature, maternity care is intimate and necessarily crosses a woman’s body boundaries at some point [3]. The potential for maternity care to compound the suffering and trauma of women who have been sexually abused is well recognised [4, 5].

Successive studies have shown that approximately 20 % of women have experienced some form of CSA [6, 7] but few women disclose it to healthcare professionals [8–11]. It is therefore likely that midwives will encounter women with a history of CSA without realising it. There is lack of clarity in the literature about the relationship between childhood sexual abuse and birth outcomes. Abuse in childhood has been associated with increased reporting of common complaints during pregnancy [12] and with premature contractions, cervical insufficiency and premature birth [13]. However, a systematic review [14] failed to find a significant association between past childhood sexual abuse and complications during the birthing process. Corroborating this finding, a case–control study by Nerum et al. [15] found that despite increased obstetric risk factors at onset of labour, women with histories of CSA were no more likely to have operative births than other women. Qualitative studies have found that most women with a history of childhood sexual abuse have apparently normal, uncomplicated births [4, 10, 16]. So women with a history of CSA do not necessarily present differently in clinical encounters from other women and may not be distinguishable during their maternity care.

Although some women with histories of CSA experience childbirth as healing [9, 11], it has been suggested that many emerge from maternity care feeling that they have been violated again [4]. Powerlessness and lack of control are important contributory factors [4, 8, 16]. In a qualitative study of women who had been raped as adults, Halvorsen et al. [17] characterised the experience of birth as ‘being back in the rape’. Leeners et al. [14] recognized the potential of both body changes during pregnancy and the actions of carers to trigger memories of childhood abuse, but noted that data on this subject are rare.

The feminist narrative study from which data for this paper were taken explored the impact that having been sexually abused in childhood had on the maternity care experiences of adult women [10]. Through the narratives of self, relationship, context and the childbirth journey recounted by women, an overarching theme of ‘silence’ emerged that created a particular challenge for those providing care for them. ‘Re-enactment of abuse’ emerged as a key theme in women’s narratives of relationship, which focused on the way women related to others and in turn received others’ attempts to relate to them. It demonstrated how, irrespective of whether women received sensitive care, their maternity care experiences could be reminiscent of childhood abuse leaving them feeling unsafe and vulnerable. The current paper explores this in more depth and concludes by suggesting strategies that healthcare professionals can employ to facilitate women’s safe passage through pregnancy and birth.

## Methods

Data were collected via in-depth interviews with women, review of their maternity care records and semi-structured interviews with maternity care professionals. Ethical approval was gained from Southampton and South West Hampshire Research Ethics Committee B.

As described elsewhere [10], the study was devised in collaboration with CIS'ters (a national charity supporting women who were sexually abused, raped and/or sexually exploited as girls by a member of their immediate or extended family), health care professionals and a local Rape Crisis Centre. Potential participants who met the inclusion criteria were identified and given details of the research by one of these agencies who were therefore gatekeepers to the study. All the women had accessed maternity services in one NHS Trust in the south of England. To be eligible they must have experienced maternity care within the local maternity service (so that if they gave permission, their records could be accessed), had some form of unwanted sexual exposure that began prior to their sixteenth birthday and been over eighteen years old at the time of interview.

Women who were interested in participating returned a reply slip to the researcher, which included preferred means of communication (i.e. telephone, email). They were contacted to discuss the study further and arrange a mutually convenient time for an interview. Of the 160 research packs left with the agencies, 23 were distributed to eligible women. Ten women returned reply slips, all of whom initially agreed to meet the researcher, but one later withdrew as she felt she might have been too upset by an interview.

Due to challenges experienced with recruitment, interviews took place over a fairly long period between November 2008 and March 2011. They were approximately 60–90 minutes long but one lasted nearly four and half hours and took place on two separate days at the woman’s request. Interviews were held in a safe, private location of the woman’s choosing. Written consent was obtained prior to the interview and as part of the process, women were asked about their sources of support. These were recorded on the consent form and a copy was given to the woman. Interviews were digitally recorded and transcribed verbatim by the researcher (EM) who was the only person both to know the identity of the women who participated and to listen to the recordings. All of the women chose the pseudonym by which they are known in the study. In keeping with narrative methodology, women were asked a broad opening question: “I’d like to find out about what being pregnant and having a baby was like for you. Starting however you want, please could you tell me?” They then recounted their experiences as they chose. All of the women spoke freely so prompts to encourage them if they were uncertain how to begin were not needed. However occasional clarification or further detail was requested from them to aid understanding.

Data from the interviews with women were analysed using the Voice-Centred Relational Method [18, 19] which involved repeated readings of the transcripts to listen for the different voices with which women speak. Initial readings were for the plot and the researcher's response, the ‘voice of the I’ (how women spoke about themselves), relationships and the socio-cultural context. Subsequent thematic analysis was conducted to facilitate synthesis of individual narratives and their translation into a more readily accessible form. This involved a further formal reading of the transcripts in which codes were identified, labelled and then organised into themes. More detail on analysis is provided elsewhere [10, 20]. Analysis was conducted by one author (EM) but discussed in detail with the others. The research team comprised a lecturer in midwifery (EM), an experienced qualitative researcher (CP) and a consultant midwife (JR).

## Results and discussion

Nine women aged 28–52 participated in the study. At the time of interview their children ranged in age from nine weeks to 28 years and some women were therefore recalling events that had occurred many years prior to the interview. All were white, British and reported heterosexual relationships, so lack of diversity is a limitation of the study. The recruitment strategy employed was important for protection of the women, but meant that all those approached had disclosed their abuse to someone and this made them atypical as many women do not disclose. Only three women had disclosed their abuse to the healthcare professionals caring for them. Irrespective of whether they had disclosed, most of the women found aspects of their pregnancy and birth reminiscent of abuse – even with the support of a trusted midwife. ‘Re-enactment of abuse’ was experienced in a variety of ways. These ranged from physical acts such as vaginal examinations that can be triggering for some women, through more nebulous similarity in bodily sensations which transport women back to their abuse, to perceived issues of power and control that leave women feeling scared and vulnerable as they did during their abusive childhoods. Sometimes these issues were anticipated by women and sometimes they were not. For this paper, they have been grouped under five headings: intimate procedures, pain, loss of control, encounters with strangers and unexpected triggers.

### Intimate procedures

Procedures that are routine for staff may be ‘strange, frightening intrusions’ for women [21]. The vaginal examination is an obvious example of one such procedure that might be expected to be difficult for women with a history of abuse. Nevertheless the literature abounds with incidences where healthcare professionals have been insensitive in conducting vaginal examinations – even sometimes when a history of abuse was known to them [22–24]. Simkin [25] believes that invasive procedures are likely to be difficult for adults when their body boundaries were not respected as children. In this study, Sally was clearly upset by an examination she had during her second pregnancy in which a swab was taken.

*But then*, *I couldn*’*t get why*, *why*, *you know*, *why do you have to keep on touching me*, *why* … *in those* – *private parts*. (Sally)

Sally linked being touched with her abuse and was surprised that this was still an issue for her even though she had already given birth once. Sue’s response to the examinations she had in the course of her induction of labour with her first baby was similar:

*and I just can*’*t cope with it at all*, *I really can*’*t and I know people were saying* ‘*well you*’*re having a baby*, *you*’*ve got to deal with things like that*, *but I couldn*’*t*, *at all*, *I really couldn*’*t* (*intake of breath*) *and um*, *I don*’*t know* – *it felt basically as if every person that came in that room did an internal and that was horrible* …*it was horrible and I* – *how it makes you feel*… *is how it made you feel when you were a child. It really does*… (Sue)

The staff with whom Sue came into contact did not seem sensitive to the fact vaginal examinations may be difficult for some women.

Two other women, Elizabeth and Mia also mentioned vaginal examinations as triggers but there was a very specific issue for Mia. In order to be gentle, some midwives insert one finger first and then another at the start of an examination [26]. Unfortunately in doing this, Mia’s sensitive midwife exactly replicated what her abuser had done.

### Pain

Women were fearful not only of the intrusion of vaginal examinations but also the pain that for some accompanied them. There were a number of other examples of pain being reminiscent of abuse. Sally’s second birth for which, unlike her first, she did not have an epidural, was very difficult. The pain of her abuse and the pain of birth seemed inextricably linked in her mind:

*and* … *it*’*s that pain*…. *I*’*ll never forget that. It*’*s*, *it*’*s* …*it*’*s horrible. You don*’*t forget it. And that*’*s the same as when you*’*re having your baby. You*’*re going through pain*, *although it*’*s*, *it*’*s gonna be a good result*, *it*’*s still*… *you still remember that* – *I can*’*t* – *you can still remember that pain*. (Sally)

Mia dreaded vaginal pain because she was worried about her abuse ‘getting into [her] head when [she] was giving birth’ and consequently she became very anguished during the late first stage of labour when she was feeling pressure from her baby’s head. She links the pain she was experiencing and her need for an epidural with feeling scared:

*I don*’*t know if it is normal to have a pain* (*laughing*) *in your whole body*, *whether it is that painful*, *but it was like absolutely horrendous so I needed to have that epidural cos I was just scared*. (Mia)

Jane had no memory of her abuse at the time of her pregnancies and only recalled it when she subsequently left her abusive husband. Nevertheless her body remembered [27] and with hindsight she realised:

…*the first sort of like connection if you like with any previous history was*, *was* … *when I was actually in second stage and the pain* - *and I just stopped breathing. You know*, *I*, *I*, *I didn*’*t stop breathing completely*, *I*, *you know in the sense that it was a medical emergency*, *but I*, *I had this*, *this*, *I could hear the midwife sort of saying* ‘*Will you please breathe in*!’

She went on to explain:

*it*’*s almost like when things are so similar*, *even if you don*’*t know that there*’*s a connection*, *it*’*s almost like your body makes it anyway and you and*, *and it takes over*. (Jane)

Although it was not the case for any of the women in this study, for some, such experiences during childbirth trigger conscious memories of abuse for the first time [28].

So far the examples provided have demonstrated the way physical similarities can make the experience of birth reminiscent of abuse. The next sections present evidence that less tangible aspects of being pregnant and giving birth can also re-enact abuse.

### Loss of control

For some women pregnancy itself challenged their feelings of integrity. While pregnant, another person (in this case the baby) was controlling their bodies as had happened during their childhoods. This could be very difficult even if the baby was planned and much desired:

*and I just felt a bit* – *again*, *out of control. This baby was taking over*. (Helen)

*I felt very much trapped*, *I*, *I think that would be a very consistent theme of being*, *hav*… *being in labour*, *um* – *possibly during the pregnancy as well*, *of this feeling of being controlled an*’ *an*’ *being manipulated by other people and not feeling like I could do what I wanted to do*. (Sam)

Having felt trapped during pregnancy, Sam had the same experience breastfeeding. She felt that:

…*it*’*s like it*’*s happening again because you are being controlled by another person. And even though I really tried not to feel like that*, *it happened every single time um that I tried breast feeding. I felt that immense feeling of being controlled by someone else*. (Sam)

Control is known to be of paramount importance to women with a history of CSA [4, 8, 9, 11, 16, 22, 29–31] and in this study women found lack of control during maternity care distressing. One aspect of Sally’s childbirth experience that she found very difficult was having some retained placental tissue removed under anaesthetic following the birth of her second child. She attributed her distress over this to lack of control during the procedure and linked it to her childhood:

…*you know you*’*ve got to go to theatre and you know*, *you know* – *what they*’*re gonna do basically*…*Um* – *maybe*, *maybe it*’*s as* – *it feels like it*, *as if it*’*s abuse again*….*Yes* – *because I wasn*’*t in control maybe. Maybe that*’*s what it was. Maybe*… *because I was*, *because I didn*’*t know what was going to happen. I didn*’*t* … *because I wasn*’*t in control when I was five. And I think that*’*s probably what it is*. (Sally)

Helen similarly suggested her need for control stemmed from the lack of it in her childhood:

*When I was a child and all this crap was happening to me*, *I wasn*’*t able to be in control. It all happened against my wishes*, *but I was a kid and it*’*s adults and you know*, *they*’*re the ones doing it* – *you don*’*t have any right* – *you don*’*t have any say really*. (Helen)

Barlow and Birch [32] argue that sexual abuse is about power, control and domination and Bass and Davis [33] recognise the damage CSA can cause to someone’s sense of control. If at a time when a child should have been gaining an understanding of her own agency or capacity to act independently and make her own choices [34] she instead learnt that she was powerless, this sets up a pattern for future encounters. For women with a history of sexual abuse, it can be very damaging when these feelings are re-ignited [8, 11]. The experience of several of the women can be summed up in the words of Elizabeth whose baby had been fed formula milk on the ward without her knowledge and consent:

*My whole childhood was about everyone doing stuff to me. Never asking*, *just doing stuff to me and now someone*’*s taken my baby and they*’*ve not even asked*. (Elizabeth)

Women frequently talked of people ‘doing stuff’ to them and the parallel with their childhood experiences of abuse and the necessity of encounters with strangers was a real concern for them.

### Encounters with strangers

Although Sue wanted a baby, she did not want to be pregnant because:

*I knew that I was going to have to let other people into my life that I don*’*t like doing*, *really*. (Sue)

The child-like voice Sam used when contemplating contact with other people exposed her vulnerability:

*I think I was conditioned right from an early age*, *so that programming*’*s always gonna be inside me and when someone*’*s either like threatening my personal like intimate spaces or hurting me*, *or telling me to do stuff and I*’*m like feeling threatened*, *I will do exactly what they say. I will be the best patient they can possibly have. I*’*ll be that star patient. But I*’*m not. I*’*m actually screaming inside. I*’*m like absolutely terrified. I*’*m expecting them to hurt me. I*’*m being good because I don*’*t want them to hurt me anymore*. (Sam)

Sam’s words are echoed in other studies where women were reported adopting child-like voices and promising to ‘be good’ so that the hurt will stop [30]. She felt submissive towards people she did not know:

…*if I*’*m with someone who I don*’*t know*, *and is a stranger and probably medical and possibly could have potential power*, *massive power over me and my life*, *I will be very*, *very um* … *good y*’*know*? *Um*, *I*’*ll be the best I can be* (*very quietly*) *cos I don*’*t want* '*em to hurt me*, *y*’*know and it*’*s so scary*… (Sam)

She was frightened by many of her encounters with medical staff. In the extract below, she describes having an epidural:

*Cos all they do is come in*, *like mess*, *fiddle with you*, *do things to you and then they don*’*t really tell you what they*’*re doing*…*and then they disappear again and leave you and it*’*s almost like you*’*re sort of waiting for the door to open. You don*’*t know who is gonna come through*, *what*’*s gonna happen*, ‘*n it is very*, *very frightening*. (Sam)

A particular issue during the citing of epidurals was positioning - many women with abuse in their histories cannot tolerate having their back to the door [28]. Uncertainty as to who was coming through the door increased women’s fear. Sue mentioned this anxiety in her interview. She had been admitted to an antenatal ward in which there were several beds in the same room and noted:

*I*, *I*’*m completely scared of the dark and to lie there with someone walking into your little curtain bit and turn the light off was horrible because you can hear the footsteps coming* … *and*, *and you know*, *footsteps have a real big meaning when you*’*ve been treated not well as a child. And those footsteps* – *you just don*’*t know who they are until they come round the curtain and um and then switch the light off and you*’*re so scared*… (Sue)

These examples demonstrate how clinical encounters can trigger memories of abuse in ways that may not be immediately obvious to those caring for the women. There are also times when the trigger is unexpected by the woman herself.

### Unexpected triggers

Being confined by an epidural was one such trigger:

*If I*’*m stuck on a bed*, *an*’ *I can*’*t get out*, *that is just like horrible it is*, *and then people coming in the room all the time and it*, *it triggers flashbacks*. (Sam)

Even though epidurals had been requested by women, the unanticipated consequence transported women back to their abuse. Sam went on to say:

…*and it makes me be the kid again* (*hesitatingly*) *an*’ *I*’*m like* ‘*oh no*! *It*’*s happening again*! (Sam)

Mia was relieved after having her epidural, but had been unexpectedly distressed earlier in her labour at home:

…*and I could feel the feeling to push and then she did an internal examination then it gave me like a bit of a problem in my head and she got a torch out*, *which is what he used to do. He used to get like a torch out to look cos it used to be like in a den*, *sort of thing*, *so*, *so she got a torch out and it was dark and I started thinking I needed to go to the hospital*. (Mia)

This distress was compounded when, awaiting the epidural in a birthing pool. Mia’s birth partners attempted to keep her safe by restraining her at the shoulders, again mirroring the actions of her abuser:

*in that birthing pool it was absolutely horrendous cos I*’*d never really had nightmares about it or anything really but in that birthing pool I could see his face*, *which I couldn*’*t really remember and how he looked and everything like he was on top of me in that pool and like I*’*d floated out of the situation that I was in and I was seeing it and nobody else was like around*. (Mia)

Mia was glad that her midwife knew her history because otherwise she feared that people would think she was just ‘kicking off’ for no reason. Sue’s flashbacks could be triggered by things that she knew might appear petty to others. She gave the example of someone sitting in a chair next to her in a crowded space:

*I had to go up to the day unit in* [*the hospital*] *quite a few times with my third and it was always really busy and I*, *I had to sit down and someone else would come and sit it in the chair next to me and that used to freak me out because I don*’*t like people that close*. (Sue)

Many of these events are specific to individual women, unpredictable and not necessarily avoidable. The experience of re-enactment of abuse will depend on the woman, how she is at that particular time, her relationship with those providing care, their attitude and the context. For these reasons, it is not appropriate to make specific recommendations from this study. Nevertheless, with insight and forethought healthcare professionals can minimise the negative impact of care on a woman and strategies are discussed below.

### Caring for women with a history of childhood sexual abuse

This paper demonstrates the ways in which pregnancy, birth and maternity care can re-enact abuse for women who have experienced CSA. ‘Re-enactment’ occurs not only as a result of events that involve the crossing of a woman’s body boundaries but also as a result of more subjective factors that relate to her sense of agency. The former are the result of overt aspects of care for which the healthcare professional’s approach can make a significant difference to the impact on the woman. An example is the vaginal examination, the most intimate of examinations [35], which is very much part of maternity care. However vaginal examinations were not universally difficult for women in this study. They could cope if they trusted those performing them and if they retained control over the conduct of examinations. This suggests that clinical procedures are not necessarily problematic in themselves but how they are conducted is important [4]. Demonstrating respect and enabling women to retain control is crucial in providing sensitive care.

It is more difficult for healthcare professionals to mediate their impact on other aspects of care that might re-enact abuse. Rather than being the result of an external action, the impact of these aspects comes from the internal associations for the woman. Reactions to pain cited in this paper exemplify this. Some women anticipated difficulty, others did not. In Jane’s case, it was only with hindsight that she was able to understand her reaction to the pain she felt. However, the fact that body memories may make a woman react in the same way she did to her abuse is reported elsewhere [25].

Re-enactment occurs whether or not a woman has disclosed her abuse and is not necessarily prevented by ‘sensitive’ midwifery, as demonstrated by the examples provided from Mia’s narrative. As only three of the participants had disclosed abuse to their midwives, most of those caring for the women in this study were unaware that they had a history of CSA and there was little in the women’s presentation to alert them to it. Midwives therefore face a silence that presents challenges in caring for these women [10] yet the relationship between midwife and woman is key. Women want to feel safe during their maternity care, and for women with a history of CSA, that means not being reminded of their abuse [29]. Control is paramount in prevention of the re-enactment of abuse [29] and this study has confirmed that anything that hampers a woman’s sense of control can be reminiscent of abuse – even the pregnancy itself. Healthcare professionals need to be sensitive to the fact that even wanted pregnancies can be difficult and challenge a woman’s sense of integrity if as a child they experienced another person taking control of their body.

Trusting relationships nurtured by open communication are crucial in helping women to feel safe. Getting to know women may alert midwives to cues that suggest that all is not well, even if the cause remains hidden. The women in this study wanted their distress to be acknowledged, but did not necessarily want their abuse to be named. The benefits of midwife-led continuity models are well recognised [36] and may help to promote the trusting relationships that are so important for women with a history of CSA.

As demonstrated by the women’s ‘encounters with strangers’ described in this paper, disempowering interactions transport women back to their childhoods and leave them feeling very vulnerable. It is therefore imperative that women are heard and respected. Sometimes this will require additional sensitivity to unspoken messages and empathy to women who may themselves be confused or distressed. As some women may not remember their abuse [4, 16] and for many others it will remain hidden, ‘Universal Precautions’ [8] are required to mitigate trauma.

## Conclusion

This paper has shown that maternity care can be traumatic for women with a history of childhood sexual abuse. However childbirth may also lead to growth and healing. Women who are not reminded of their abuse can ‘feel safe’ [29] but delivering this level of care is challenging. Advice that ‘the most useful guide to providing appropriate care for a woman with a history of abuse is the woman herself’ is sound [4:89] and providing care that leads to healing rather re-enactment of abuse must be the aspiration of every maternity care professional.
